# Personality Cult or a Mere Matter of Popularity?

**DOI:** 10.1007/s10767-022-09423-0

**Published:** 2022-05-04

**Authors:** Anne-Mette Holmgård Sundahl

**Affiliations:** School of History Philosophy, Politics & International Relations, Victoria University of Wellington, PO Box 600, Wellington 6140, New Zealand

**Keywords:** Personality cult, Charismatic authority, Max Weber, Celebrity

## Abstract

This paper introduces a theoretical model for distinguishing between mere popularity and personality cults as there currently is an inflated use of the personality cult concept, especially in news media, attaching it to significantly different phenomena. The model is based on Weber’s concept of charismatic authority and consists of three parameters, widespread symbolic elevation, resilience and religious parallels, covering a representational and social practice dimension. Both dimensions are needed to constitute a personality cult. Trump, Putin and Ardern are used as examples of the model’s ability to distinguish between cult and non-cult phenomena. The comparison shows that only Trump and Putin have a cult on both dimensions. Mere popular politicians like Ardern are more comparable to celebrities as these do not have the same authority and power over the followers as leaders with a personality cult – despite potentially showing some cultlike tendencies on the representational dimension. Popular politicians are thus especially characterised by lacking the key social practice aspect of personality cults. As they might still exhibit some cultlike characteristics, the different phenomena are best perceived as being on a continuum ranging from mere admiration or popularity to a personality cult.

## Introduction

The fascination with Vladimir Putin’s machoism has revived interest in personality cults, understood as the exaltation of an individual’s authority through the creation, projection and spread of a godlike image paralleling religious worship (Pisch, 2016: 53–54). It is, however, not only non-democratic, neo-Soviet leaders like Putin who are said to have a personality cult. Commentators argue that Donald Trump has a ‘cultish quality’ which Trump acknowledged by saying he could ‘stand in the middle of Fifth Avenue and shoot somebody’ without losing voters (Aslan, 2017). Although Trump’s presidential period arguably had some degree of democratic backsliding (Ingraham, 2020), and he can be considered an extreme case (Nai et al., 2019), even a relatively more well-regarded leader like New Zealand’s Prime Minister Jacinda Ardern has allegedly acquired ‘a global cult’ (Ganesh, 2020). But can all three political leaders be said to have personality cults? They have different communicative styles, images and personalities – and different degrees of divisiveness. What distinguishes having a personality cult from ‘just’ being popular?

As limited scholarly attention has been given to how to systematically distinguish between personality cults and other forms of popularity, like a celebrity (Hendriks, 2017: 347), the paper focuses on Weber’s concept of charismatic authority and how related studies on celebrities and fandom have used this concept. Charisma is particularly useful as a starting point since it concerns how authority is constructed and can thus be used to ‘unpack the appeal’ of the cult (Cocker & Cronin, 2017: 455). Celebrities constitute the contrast category since these phenomena resemble popular politicians without a cult. Both phenomena can have a fan following and enjoy a somewhat elevated status, but celebrities do not gain additional personal power. Importantly, celebrities and popular politicians do not exhibit the high levels of charismatic authority that define personality cults. Charismatic authority is constructed through images, but it requires an additional layer to be classified as a personality cult – like participation in rituals and sycophantic behaviour by devoted followers. Different things go into constructing celebrity and charisma, respectively.

I will thus critically appropriate Hendriks’ (2017) model of the ideal–typical celebrity and charismatic to introduce my own comparative model. Hendriks’ model has been chosen as a starting point as it systematically separates celebrity from charisma and remains consistent with how charismatic authority was originally conceptualised by Weber. The model consists of three parameters covering a representational and sociological dimension. The parameters are whether (1) the symbolic elevation is widespread at different levels of society, (2) the representations of the leader and associated practices of interaction with them are contingent on his or her political success (“resilience”) and (3) the leader representations and associated practices of interaction with them have religious parallels. Based on these parameters, the phenomena observed can be placed on a continuum ranging from mere admiration to a fully fledged personality cult. The purpose of this paper is thus to formulate a theoretical model that is empirically applicable and can aid in separating seemingly similar phenomena. Following the introduction of the theoretical model, the alleged cults of Putin, Trump and Ardern are analysed as examples of the model’s ability to distinguish between phenomena. These examples are not comprehensive and are purely intended to be indicative and illustrative. These three politicians are useful examples as they are contemporary leaders of different genders, with different leadership styles, and representing different regimes (democracies and autocracy) and government types (presidential and parliamentarian). These differences will help to demonstrate the model’s empirical applicability.

### Conceptualising Charismatic Authority

Plamper defines personality cults as the ‘godlike glorification of a modern political leader with mass medial techniques and excessive popular worship for this leader’ (Plamper, 2004: 33). Elsewhere, he states that a personality cult at its most basic level can be defined as ‘the symbolic elevation of one person much above others’ (Plamper, 2012: xv). It is worth noting the use of the words ‘godlike glorification’ and ‘worship’ that carry religious connotations. Plamper states that as a person gets elevated, the individual is endowed with ‘sacrality’ or a ‘sacral aura’ (Plamper, 2012: xv). This relates to Weber’s charismatic authority which is a ‘personal devotion to, and personal trust in, revelations, heroism, or other qualities of leadership in an individual’ (Weber, 1994: 312). Charisma is ‘a certain quality of an individual personality by virtue of which he is considered extraordinary and treated as endowed with supernatural, superhuman, or at least especially exceptional powers or qualities’ (Weber, 1968: 241). Although charisma is a widely used concept in personality cult studies, it lacks a clear definition and has acquired the status of a residual category, capturing what cannot be labelled as rational-bureaucratic or traditional authority in Weber’s tripartite classification of authority (Leese, 2014: 349–350; Hendriks, 2017: 350).

The lack of a clear definition is reflected in the application of the concept to several different, but related, phenomena, making it difficult to separate them. Wedeen comments that scholars studying personality cults ‘often argue that “successful” rhetoric and symbols operate to produce “legitimacy,” “charisma,” or “hegemony,” thereby enabling political leaders to win support for themselves and their policies by fostering collective ethnic, national, or class identifications’ (Wedeen, 1998: 505–506). This characterisation does not, however, reveal the idiosyncrasy of personality cults. Symbolism and rhetoric are something all political leaders use – albeit to various extents and taking different forms. So, when can they be used to produce the charisma associated with personality cults? Commenting on Syria, Wedeen acknowledges this difficulty in differentiating between phenomena by stating that what might be perceived as ‘a charismatic, loyalty-producing regime’ à la Weber is in fact ‘its anxiety-inducing simulacrum’ (ibid.: 506). Leese similarly acknowledges that it can be difficult to distinguish between ‘nascent’ and fully fledged personality cults. He uses Putin as an example of such a border case but without specifying how Putin may come short of a fully fledged cult (Leese, 2014: 342). Despite scholars acknowledging the difficulties in clearly defining and separating personality cults from ‘simulacrums’, no solution is offered to rectify this identification problem.

The dilution of the charisma concept is, however, most evident in the way it has been applied to the study of celebrity cults. Marshall, for instance, argues that ‘in contemporary culture there is a convergence in the source of power between the political leader and other forms of celebrity’ (Marshall, 1997: 19) with the ‘enchantment and allure’ associated with charismatic authority extending from the political culture into popular culture (Marshall, 2019: 91). Ravid and Currid-Halkett equally argue that ‘celebrities exhibit qualities of “charismatic authority”’ (Ravid & Currid-Halkett, 2013: 186), while Cocker and Cronin use charismatic authority to describe the appeal of British YouTube personalities, illustrating the use of the concept in a non-political setting. Cocker and Cronin acknowledge that the concept originated in the political and religious arenas, which are institutional fields with ‘mass reach, ample resources and legitimate power’ (Cocker & Cronin, 2017: 456) – implying that these are areas in which political personality cults and celebrity differ. Charismatic politicians thus have legitimate power in a way celebrities cannot have. However, despite these remarks, Cocker and Cronin do not justify using the concept of charismatic authority on celebrities. To fully understand how the two arenas of personality cults and celebrity differ in the exercise of authority, further conceptual clarification is needed.

### Differentiating Charisma and Celebrity

Attempting to clarify the charisma concept, Hendriks outlines nine characteristics of the charismatic ideal type, emphasising how these differ from celebrities that are frequently described as charismatic in a way that is inconsistent with the Weberian concept. The ideal–typical charismatic claims/is perceived to have ‘transcendent knowledge’ and ‘a unique higher mission’. Because of these extraordinary abilities, the charismatic attracts ‘followers’ and inspires people to accept his or her authority as ‘an ethical duty’. He or she further ‘transcends everyday economic life’, ‘unleashes revolution upon history’ and ‘is only peripherally tied to media’s power in society’. The source of legitimate authority relies on the followers’ ‘belief’ in his or her charisma, which also means that the charismatic is ‘vulnerable to delegitimizing crises’ (Hendriks, 2017: 361).

Unlike the ‘exceptional qualities’ of the revolutionary charismatic, Hendriks’ ideal–typical celebrity is defined by possessing ‘media prominence, not transcendent abilities or any special form of knowledge’ – thus making it possible for the celebrity to serve as an object of psychological identification, appealing to fans for being 'just-like-us' (ibid.). Turner similarly argues that television shows have shown that there is no need ‘to establish the individual’s ability, skill, or extraordinariness as the precondition for public attention' (Turner, 2013: 10). It is perhaps for this reason that most definitions of celebrity remain somewhat general and superficial. Rojek defines it as ‘the attribution of glamorous or notorious status to an individual within the public sphere’ (Rojek, 2001: 10), without specifying where this status originates. Turner equally downplays the importance of special skills or talent by arguing that a public figure becomes a celebrity when aspects of their private life overshadow their public role, like sports achievements (Turner, 2013: 8). In a similar vein, Ravid and Currid-Halkett emphasise how celebrities are ‘individuals who share the distinguishing trait of being frequently visually documented by the media and popular press with other highly visible individuals at high-profile events' (Ravid & Currid-Halkett, 2013: 183). Instead of being rooted in great achievements, celebrities’ ‘capacity for fame’ is contingent on how well they differentiate themselves from their competitors (Turner, 2013: 5).

This is closely connected with Hendriks’ view of celebrities as being part of the machinery of commercialism. As such, the celebrity is unlike the charismatic, not a revolutionary figure but instead entrenching the status quo and taking part in ‘the media’s attempt to monopolize symbolic power’ (Hendriks, 2017: 361). The celebrity does not rely on a consensus of belief and is not vulnerable to delegitimizing crises as interest is not diminished by the ‘demystifying awareness of the celebrity system that turns people into “stars”’ – contrarily, ‘charisma-undermining dynamics’ are, according to Hendriks ‘essential’ in celebrity culture (ibid.: 358).

Commenting on Hendriks’ concern with the inappropriate use of charisma when describing celebrities, Nixon argues that this expresses a ‘historic definition’ that does not account for the change in the meaning of language over time (Nixon, 2021: 175). Nixon thus prefers the more ‘modern’ conceptualization advocated by Allison and Goethals in which charisma is a ‘personal quality attributed to those who arouse fervent popular devotion and enthusiasm’ or ‘personal magic of leadership arousing special popular loyalty; a special magnetic charm or appeal’ (as cited in Nixon, 2021: 175). While Nixon does not elaborate on in what way he considers Allison and Goethal’s definition more modern, his rejection of the Weberian ideal as presented by Hendriks is indicative of how different phenomena are confused due to lack of conceptual clarity. By choosing ‘modern’ conceptualizations that align more with the popular use of the concept by, for instance, the media, distinguishing the separate and distinct phenomena of celebrity and personality cults becomes difficult, if not impossible. Marshall noted that celebrity is an ‘encompassing term’, whereas concepts like ‘hero, star, and leader […] relate to specific functions in the public sphere’ (Marshall, 1997: 7). With this in mind, a charismatic leader can be a celebrity, or in other words popular, but not all celebrities are charismatic.

### Can Celebrities have Authority?

Furedi nonetheless argues that there is a ‘tendency to outsource authority to celebrity […] to bypass the problem of legitimacy by politicians and other figures’ (Furedi, 2010: 493). The traditional authority of the political elite is eroding and being replaced by celebrities. While Furedi acknowledges that celebrities might not possess the heroic or magical qualities of charismatics, and that they often ‘appear as the very opposite of this Weberian ideal’, he maintains that their authority lies in them serving as role models (ibid.: 495). As an example, Furedi mentions the British chef Jamie Oliver as someone ‘endowed with prophet like status and assigned the role of saving the nation’s children from the scourge of junk food’ – even the Prime Minister and the Queen reported that they ‘had seen the light’ (ibid.: 496).

While celebrities can certainly be role models, it can be questioned whether this translates into actual authority. Paraphrasing Weber, Spencer argues that charismatic authority is ‘the purest form of authority in that it claims the right to break through all existing normative structures’ (Spencer, 1970: 124–125). In the case of Jamie Oliver, he allegedly broke through the existing norms pertaining to the food served at schools in the United Kingdom and created a new norm – to eat healthier. But does this make him an authority? It was reported that although Jamie Oliver campaigned to change eating habits, many schools continued to serve high-fat and sugary foods for lunch (Rose, 2019). Even though Jamie Oliver was a role model, he lacked the authority to *command* obedience in the absence of a willingness to do so. In a Weberian sense, authority is ‘the probability that a command with a specific content will be obeyed by a given group of persons, *despite resistance* [italics added], regardless of the basis on which that probability rests’ (Uphoff, 1989: 300–301). The special position of authority further permits the issuer of the commands to back them with rewards or sanctions (ibid.: 301). This thus relates to Cocker and Cronin’s allude to the political arena’s legitimate power. Jamie Oliver’s campaign was not entirely successful due to his lack of ability to sanction non-compliant schools. While celebrities can *inspire* people to act in a certain way, they cannot *enforce* it. This is supported by Hendriks, who maintains that celebrities cannot have charisma in the Weberian sense since it is a form of authority and not merely the ability to influence people. Celebrities cannot, unlike charismatic politicians, issue authoritative demands (Hendriks, 2017: 350–351).

### Ideal Types and the Matter of Empirical Applicability

However, although Hendriks’ separation of the two ideal types is appealing, it is less applicable empirically precisely because it focuses on the *ideal* in its purest form. Undertaking a less rigid approach, Street (2004) examines the phenomenon of the celebrity politician and identifies two variants of the phenomenon: elected politicians using ‘celebrityhood’ as a strategic tool and celebrities using their popularity politically. The first mentioned type of celebrity politician comes in two variations: the politician who capitalises on a background in entertainment, showbusiness or sport, and the politician who uses techniques borrowed from celebrities to further his or her image (Street, 2004: 438). Unlike Hendriks’ strict separation of the two phenomena, Street thus perceives them as intertwined. This makes Street’s approach more useful when explaining, for instance, Trump, since his being a public figure prior to getting elected president has influenced his image significantly. Hendriks acknowledges that the phenomena are inseparable empirically by stating that self-help coaches, gangsta rappers and contemporary demagogues have a ‘hybrid habitus’ that is only comprehensible when considering the tension between the two ideal types (Hendriks, 2017: 361–362). Hendriks further asserts that ‘contemporary demagogues supplement the traditional, politically instrumentalized charismatic claim with a controversy-fuelled celebrity status’ (ibid. 363). Using Trump as an example, he argues that the negative attention during the presidential campaign gave Trump more media attention than Hillary Clinton, which ultimately confirmed his charisma to some supporters (ibid.). This departs from Hendriks’ ideal type, where charismatics are vulnerable to delegitimizing crisis and stand outside of the media. Celebrities are, contrarily, dependent on media attention and live by the mantra ‘there is no such thing as bad publicity’ (ibid.: 361). Trump thus managed to assert his charismatic claim through the same dynamics that, according to Hendriks, govern the celebrity ideal type, confirming that it is unlikely that any empirical cases will fit neatly into the two boxes and correspond to the characteristics set up by Hendriks. This corresponds to Abdulaev and Shomron’s characterisation of Trump as a celebrity politician (Abdulaev & Shomron, 2020: 5), although their categorisation equally does not distinguish celebrity from charisma. When trying to develop a model for comparing different phenomena on the spectrum between mere popularity and having a personality cult, the question is whether a phenomenon can be considered a personality cult if it does not correspond to Hendriks’ Weberian-based ideal. And if no empirical cases live up to the ideals, how do we then categorise and systematically compare the intermediate phenomena? Hendriks’ ideals can establish the extremities of the spectrum, but not delineate when empirical phenomena transition from mere popularity to a personality cult.

In sum, while Hendriks’ model clarifies Weber’s charisma concept, it does not introduce any operationalizable measures and it does not distinguish between degrees of charismatic authority. While Hendriks’ approach is less applicable empirically, Street overlooks that celebrities cannot have legitimate authority and that celebrity cults do not have ‘mass reach, ample resources, and legitimate power’ like political cults – the two phenomena might be intertwined but they are not the same. Both Street and Hendriks’ models treat charismatic politicians or politicians using elements of celebrityhood as a uniform group when compared to a group from a different sphere, namely celebrities. Street would presumably categorise Ardern, Trump and Putin as celebrity politicians, while they would fall somewhere between Hendriks’ two ideal types – without it being possible to categorise their differing hybridity. Consequently, these approaches do not make it possible to distinguish these three leaders, although most observers would agree that they differ in significant ways. A theory focusing on how different popular political leaders relate to one another and exhibit different degrees of charisma is thus needed.

#### Introducing a New Comparative Model

This section introduces a model that can be used to distinguish between different degrees of charisma. The model’s three parameters cover two dimensions: cults as representations of the leader and cults as social practices. The former deals with how charismatic authority is constructed, mainly through images, while the latter concerns how these images are used. Reiterating Plamper’s definitions, ‘symbolic elevation’ and ‘godlike glorification’ are mainly about the representation of the leader, while ‘popular worship’ entails a religious practice or ritual, consequently making the personality cult into a social phenomenon. This is further emphasised by Morgan who states that cult construction has a ‘social nature’, and charisma is ‘conferred upon the individual through a complex social process’ (Morgan, 2017: 103, 110). Cult communicative artefacts, typically representations of the leader in the form of portraits, sculptures, poems and so on, signal publicly that others recognise the leader’s charismatic authority. Márquez notes that ‘the social recognition of charisma is, in turn, intensified the more cult communicative artefacts pervade public spaces’ (Márquez, 2020: 23–24). The two dimensions thus reinforce each other, and both are needed to constitute a personality cult.

Power is attributed to the charismatic leader through the perception of him or her being exceptional (Halley & Ordner, 2009: 59). Leader representations show this exceptionality – whether it is through godlike depictions or due to the sheer plenitude of images. Additionally, images are in themselves a political force shaping politics; they are expressions of power (Bleiker, 2018). Marin even argued that the king’s portrait is the foundation of his power as it gives him a real presence (as cited in Falasca-Zamponi, 1992: 77). Thus, by examining images, the representational dimension explores the foundation of the leader’s power as he or she is represented as exceptional. However, to ensure that the charismatic authority is embodied in the individual and not merely in the institutions – as would be the case with civil religious worship of the leader – the sociological dimension of the cult is needed. This dimension reflects the extent of the power; is the leader powerful, or charismatic, enough to make the followers obey demands or act in ways that violate laws or societal norms? The charismatic leader is considered a revolutionary exactly because he or she ‘embodies’ an authority that can ‘displace the existing legal order’ (Márquez, 2016: 11). The charismatic differs from celebrities as he or she ‘blazes new paths and opens new possibilities’ (Berenson, 2010: 25).

The model’s parameters are mainly derived from the five ways in which Plamper argues modern personality cults are distinct from the cults of the monarchs and tsars. Modern cults are characterised by (1) being products of mass politics, (2) using mass media and mass-produced cult products, (3) only emerging in closed societies, (4) being secular and (5) being patricentric (Plamper, 2012: xvii-xviii). The first, second and fourth characteristics will be incorporated into the model introduced here. The characteristic regarding closed societies will be omitted as it concerns the structure of society and political context more than the matter of images and how they are being used. In the Stalin era, the political sphere was highly hierarchical, and Stalin wielded his power in a way that made all others politically subordinate. A democratic and postmodern society will be structured differently. It is beyond this paper’s scope to examine the difference between democracies and autocracies. For the purposes of this paper, I treat them as equal since institutional constraints in the form of democracy do not impede charismatic authority (Márquez, 2016).

The characteristic regarding the patricentric nature of modern personality cults will also be omitted. Plamper justifies this characteristic by arguing that power ‘is distributed unequally according to gender, the personality cult reflects this asymmetrical distribution and represents those members of society who are most powerful – men’ (Plamper, 2012: xviii). While this might be an accurate observation when examining cults from the era of Stalin or Mao, the same is not the case in the current postmodern context. Anno 2021, ten countries have a woman as Head of State, and thirteen countries have a woman as Head of Government (UN Women, 2021). Although personality cults might be gendered, scholars should not rule out female cults – especially given that women in power have become more common. If they can hold the power of the office, they can gain charismatic authority.

### The Three Parameters of Charismatic Authority in Personality Cults: Widespread Symbolic Elevation

The model’s first parameter concerns whether there is a widespread symbolic elevation at different levels of society. By using the phrase ‘symbolic elevation’, borrowing from Plamper’s definition, both genuine worship of the leader and depictions intended to be ironic or ridiculing will indicate the cult’s reach. Symbolic elevation does accordingly not assume that depictions are exclusively positive – manipulated images of Putin riding a shark are, for instance, intended to expose the exaggerated masculinity in his official depictions, but they nonetheless still contribute to and reinforce the Kremlin’s narrative of Putin as a macho man. Under the assumption that any exposure is good exposure, both contributions that mock and bolster a certain image – for instance, Putin’s machismo – are consequently considered part of the personality cult. This is consistent with Hendriks’ observation that the negative attention Trump attracted during his presidential campaign gave him more media exposure, which in turn strengthened his charismatic claim. Admittedly, constant ridicule without venerating representations to balance it can undermine charisma. The fact that there is widespread exposure – of any kind – is an indication of a cult, but there must be some genuinely worshipping contributions to the cult for it to be characterised as such.

Furthermore, ‘widespread’ entails two things. First, that symbolic elevation is evident in mainstream media and something that most people at least know about even though they might not contribute to or participate in the cults themselves. Márquez argues that it is the widespread dissemination of cult artefacts that makes it a *social* rather than just an *individual* recognition of the leader’s charisma (Márquez, 2020: 23). Following Plamper’s first and second characteristic of modern cults, modern personality cults are ‘the children of mass politics’ since they, by using mass media techniques, are directed at the entire population. They thus reach a much larger audience than the premodern personality cults that targeted the elite (Plamper, 2012: xvii). This tendency is even more pronounced among postmodern personality cults due to the internet and smartphones making it easier to both receive and produce content online.

Contrary to Hendriks’ ideal type, I thus *do not* expect the charismatic leader to stand outside of the media – quite the contrary. While Plamper did not explicitly formulate his five characteristics with Weber’s charismatic authority in mind, the two are not incompatible. I here rely on Strong and Killingsworth’s discussion of the media in relation to charismatic authority. In line with Hendriks’ stance, Bensman and Givant argue that the modern political arena cannot induce an intimate relationship between leader and followers as it involves millions of people, while Loewenstein similarly states that true charisma cannot be founded on media exposure as ‘mass media in an open society act as disenchantments’, making the leader less magical and enchanting to the followers (as cited in Strong & Killingsworth, 2011: 399). Strong and Killingsworth, however, reject these arguments by stating that if the followers accept the leader’s charismatic claim, it does not matter how this relationship was established in the first place (ibid.: 400). This is not only a compelling argument but also a necessary assumption to use Weber’s concept in a modern – or postmodern – context.

Second, widespread entails that the elevation process, or construction of charisma, happens on different levels of society and not only among the elite. Although Weber largely considers charismatic authority a top-down phenomenon where the followers’ agency is limited to rejecting or accepting the leader’s charisma, Hendriks argues that followers can equally construct the charisma from below (Hendriks, 2017: 357, 359). For the symbolic elevation to be widespread, people without an official regime position must thus likewise contribute to the cult.

In terms of the two dimensions of this parameter, the representational aspect is operationalised as whether the cult images originated from both official and unofficial sources. If the cult images are only made by grassroots, namely, people without an official regime position, it indicates that the cult is not cultivated and encouraged by the regime. Weber considers, as mentioned, charismatic authority to be constructed top-down. Similarly, in Burns’ idea of ‘transforming leadership’, which is effectively based on the leader’s charisma, the leader takes the ‘major part in maintaining and effectuating the relationship with followers’ (Burns, 1979: 20) – thus emphasising the importance of the regime’s involvement in the cult. This top-down approach certainly overlooks the significant contributions to the construction of charisma from below. It can thus be considered a necessary – albeit not sufficient – condition to have a fully fledged personality cult that the leader actively cultivates an image of being extraordinary. By the same token, if charisma is only constructed top-down, it suggests that the leader’s charisma is rejected by the followers – the bid to establish charismatic authority was unsuccessful. Rejection of the leader’s charisma means that there is no power to be gained from cultivating a certain image of the leader. Consequently, it cannot be characterised as a personality cult. This dimension of the parameter thus reflects the ‘dialectical’ relation between charismatic leaders and followers as both ‘depend on each other’s mutual recognition in order to function’ (Halley & Ordner, 2009: 60).

The sociological aspect of this parameter focuses on the existence of sycophantic followers, meaning people who make exaggerated statements about the leader. This type of praise is more than just admiration and goes beyond what society finds acceptable. Sycophantic behaviour among fellow politicians or people with an official position in the regime is particularly significant, as the stakes are higher among the political elites. Although loyalty-signalling is equally observed among the grassroots, it ‘requires the existence of expectations of rewards or punishments for recognising the charisma of the leader’ (Márquez, 2020: 30). People are more motivated to praise the leader when career opportunities and promotions are at stake, which is mainly a concern among the leader’s closest associates. Similarly, a study of loyalty displays in China showed that junior officials are prone to display sycophantic behaviour towards their seniors despite norms against cult worship. Violating this norm publicly constitutes a costly signal which makes it a more credible sign of loyalty as the sycophant commits to the leader (Chung-Hon Shih, 2008: 1178). Since it is the political elite surrounding the leader, that is most likely to bring about his or her eventual downfall, it is equally among these people that loyalty-signalling is most important.

### The Three Parameters of Charismatic Authority in Personality Cults: ‘Resilience’

The second parameter concerns the degree to which representations of the leader and attachments to him are contingent on his successes or failures, meaning whether the alleged cult is vulnerable to delegitimizing crises. On the representational dimension, the resilience parameter covers to which degree political decisions and stances are reflected in leader depictions. If leader depictions frequently refer to policies enacted by the leader, it indicates that the leader’s popularity, or the public’s acceptance of the charismatic claim, is contingent on political success. If a policy spearheaded by the leader fails to have the intended effect, the leader’s popularity will plummet. Conversely, if leader depictions do not contain references to specific policies, it indicates the cult has become detached from the leader’s political success. Consequently, a political failure will not affect the leader’s popularity and thus not prevent asserting charismatic authority – the cult will be resilient. If the leader receives negative attention, the followers’ loyalty might even be strengthened, as it will be perceived as an attempt from political opponents to undermine the leader. This further supports the argument that even negative depictions contribute to the construction of the leader’s charisma, partly because these might infuriate genuine supporters and make them even more committed to the cult, thus confirming the leader’s charismatic claim.

The logic behind this parameter, however, deviates from Weber’s conceptualization of charisma as he states that the leader’s ‘divine mission must “prove” itself in that those who faithfully surrender to him must fare well. If they do not fare well, he is obviously not the master sent by the gods’ (Weber, 1969: 249). To Weber, acceptance of the charismatic claim is thus linked to the leader’s success. My departure from Weber in this regard is founded in the paper’s focus on stronger forms of charisma like the authority claimed by the popes when declaring themselves infallible. The dogma of papal infallibility entails that because the pope speaks in his capacity as ‘shepherd and teacher of all Christians’, papal decisions on theology and ethics cannot be questioned. The pope is infallible in that not only is he exempt from making errors – there is no *possibility* of him making any in the first place. This dogma has the advantage of making the Church’s members more compliant and loyal, as seeking change from within the Church is impossible (Ferrero, 2011: 89–91). Zacharias similarly argued that the foundation of the Communist party was Stalin’s infallibility. According to Zacharias, ‘for the Communist to hand himself over body and soul […] to the directives of the party can therefore only seem justifiable to himself, because only by following them does he think that success can be achieved’ (Zacharias, 1950: 471). Consequently, instead of political success and achievements leading to charismatic authority, as it is theorised by Weber, success is contingent on acceptance of the charismatic claim. The arrow of causality has been reversed.

While the leader’s popularity can continue to be tied to success in the public eye despite official depictions avoiding references to policy, the nature of the official depictions will to some extent affect their unofficial counterparts due to the reciprocal relation between them. For example, in his book on the ‘Hitler myth’, Kershaw argues that while the party’s popularity correlated positively with the country’s fate, this was not the case with Hitler’s own popularity as he was able to avoid responsibility and backlash when something went wrong. This observation remained true until after Germany’s 1943 defeat at Stalingrad (Kershaw, 1987: 200). This suggests that it takes a crisis of a certain magnitude or level of devastation to revoke the cult’s resilience.

This tolerance will be much lower for leaders who have no or weak charismatic authority. Burns distinguishes between two different forms of leader–follower relations, arguing that in transactional leadership, the relationship is defined by a bargaining process in which the leader is followed because he or she performs a service, like implementing a certain policy (Burns, 1979: 19–20). Similarly, Levitsky and Way argue that leaders who base their support on patronage will experience a more fragile tenure as economic crisis, domestic opposition or external pressure will lead to defection as supporters lose confidence in the regime’s ability to deliver (Levitsky & Way, 2012: 870–871). Transactional leadership is thus contrary to the previously mentioned transforming leadership, the equivalent of Weber’s charismatic authority, as the leader has a connection with the followers that goes beyond delivering a service (Burns, 1979: 19–20). It is instead based on the pursuit of a common higher purpose linked to the charismatic leader’s role as a revolutionary. The transactional leader, or the leader reliant on patronage, is thus more vulnerable than the charismatic, as his followers only follow him for their own gains.

The charismatic leader is thus tied to the followers on a deeper level, which makes the cult more resilient. In the USA, in particular, party affiliation is part of one’s identity, so people tend to stick with their political candidate no matter what (Guskin & Williams, 2017). If cult followers consider participation in the cult as vital to their identity, one could imagine that similar loyalty can be observed among cult followers. Cassiday and Johnson highlight how the Putin mania is part of a larger trend in Russia of using consumption to ‘express individual agency, construct identity, and grapple with political change’. Buying a $600 Putin t-shirt thus becomes a way of expressing personal preference and own identity (Cassiday & Johnson, 2013: 52). Followers of postmodern cults might be more motivated by the cult as a lifestyle choice than as an ‘ethical duty’ as claimed by Hendriks in his ideal typology (Hendriks, 2017: 361).

The social practice dimension of this parameter concerns whether people are willing to overlook certain aspects of the leader’s image or behaviour. Hendriks states that a charismatic has followers who are believers, while celebrities have fans that use the celebrities’ images and products as a lifestyle choice (Hendriks, 2017: 361). If the people who subscribe to the cult and accept the leader’s charisma are not just fans but followers, they will have confirmation bias in the sense that they will overlook characteristics or actions of the leader that contradict pre-existing views they have of him or her. If supporters instead are merely fans of the leader, the leader will not be without impunity, and people will openly acknowledge his or her fallibility, leading to a rejection of the leader’s charismatic claim. The ruler’s perceived infallibility most noticeably manifests itself in the followers’ will to obey a command from the leader without question. If the ruler is infallible – how can he or she be wrong in demanding something that might violate what society usually finds acceptable?

Although Weber considers charisma contingent on success, the logic of the resilience parameter is compatible with other aspects of his theory. In Hahl et al.’s (2018) study on how voters can view a political candidate positively despite him being a ‘lying demagogue’, they find that if people feel disenfranchised by the political establishment, they are more likely to consider violations of established norms, like honesty, as a sign that the candidate in question is a champion of the people. Exposing certain negative traits might thus confirm his status as a champion of the people standing outside of the political establishment. This is compatible with Weber’s idea of the charismatic leader being a revolutionary. Even if the followers are aware of the leader’s shortcomings, they might overlook them because he or she is perceived to be a revolutionary.

### The Three Parameters of Charismatic Authority in Personality Cults: Religious Parallels

The third parameter reflects whether leader representations and associated practices of interaction with them have religious parallels. Charisma, *devotion* to an *extraordinary* individual with *superhuman* powers, has since ancient times been used to describe an association with the divine – in the Apostle Paul, ‘kharisma’ refers to God’s gift of eternal life. This association continued until Weber secularised the concept (Berenson & Giloi, 2010: 3). This is further reflected in Plamper’s characteristic regarding modern personality cults being secular – the leader’s body ‘absorbs all of the sacral aura’ due to the expulsion of God from ‘society’s metaphysical space’ (Plamper, 2012: xvii). As traditional religion gets deemphasized or discouraged, people start worshipping secular figures like political leaders. Zitser argues that ‘religious allusions’ were essential in transferring the sacrality that made Peter the Great’s followers perceive themselves as ‘a secular priesthood of believers in the tsar’s charismatic authority’ (Zitser, 2018: 10). Religious parallels are thus significant in personality cults, not only because they emphasise the leader’s superhuman qualities but also because they establish a community of believers, united in worship of the leader. On the representational dimension, this parameter covers whether the leader gets depicted like a religious icon – for instance, as a saint with a halo or surrounded by shining light. If the leader is conversely depicted more mundanely or as ‘an equal’, this indicates that there is no personality cult. The social practice dimension reflects whether people participate in rituals. According to Márquez, ‘a leader cult exists wherever there are frequent, if not necessarily regular or fully formalised, rituals of worship or veneration focused on the leader or on leader-related symbols’. These rituals can be everything from everyday conversations to sports games and work meetings (Márquez, 2018: 12). If there are no signs of rituals being performed, this indicates that there are no cultlike tendencies in terms of social practice.

### Establishing a Baseline for Comparison: the Case of Stalin

Differentiating between two dimensions means that even if the alleged cult has cultlike tendencies on all three representational parameters, it cannot be a fully fledged personality cult if this is not mirrored in social practices. In such a scenario, the leader would be in the middle of a continuum between being a mere popular politician and having a fully fledged personality cult. Popular politicians without a personality cult share similarities with ideal–typical celebrities as defined by Hendriks, as they are prominent in the media and have a considerable number of fans. Conceptualising the move from being a popular politician to having a personality cult as a continuum thus reflects the interconnectedness that Street and Hendriks identified between politicians and celebrities and charisma and celebrityhood, respectively.

**Fig. 1:**
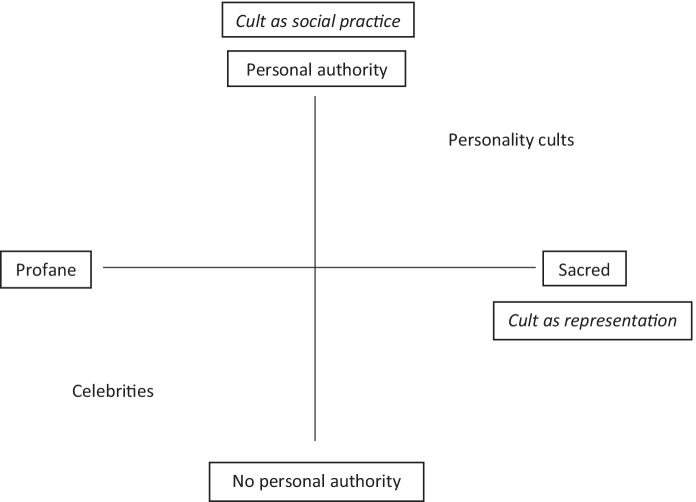
The two dimensions of personality cults and related phenomena

As there are two dimensions, it is possible to illustrate this as a quadrant (see Fig. 1). The horizontal axis illustrates the representational dimension ranging from profane to sacred, while the vertical axis forms the social practice dimension covering degrees of personal authority. Personality cults are thus in the upper right quadrant, as these phenomena exhibit cultlike tendencies on both dimensions. The Stalin cult is useful as a baseline for how phenomena in this quadrant manifest themselves empirically, as there is general agreement that Stalin, in fact, had a personality cult (Pisch, 2016; Plamper, 2012; Strong & Killingsworth, 2011). Despite commentators labelling the phenomena surrounding Putin, Trump and Ardern as personality cults, even the relatively more autocratic Russia does not constitute a fully closed, totalitarian regime on par with Stalin’s Soviet Union. While this paper does not explicitly explore the differences in regime type, using Stalin as the unambiguous baseline to assess the model’s usefulness accommodates the scholars who are not yet convinced that personality cults can come to exist in countries that are not fully closed.

Regarding the first parameter, widespread symbolic elevation, Stalin’s cult was reflected in various media, from movies, posters and photographs to paintings, poetry and other forms of crafts (Plamper, 2004: 125). There was a small catalogue of officially approved images of Stalin, constituting the basis for subsequent depictions, and artists had to submit their work for the regime’s approval. In addition to the official dissemination of Stalin cult paraphernalia, it was common to find Stalin’s portrait in private homes hung in a ‘Lenin corner’ or ‘Stalin room’ alongside icons (Pisch, 2016: 354–355). Stalin’s cult was not confined within the borders of the Soviet Union. His 70th birthday, allegedly ‘the most extensive celebration of his leader cult’, was an international event attended by notable foreign guests like Mao Zedong and Walter Ulbricht (Behrends, 2004: 161). As the cult permeated all layers of society, there was thus widespread symbolic elevation from a representational perspective. As to the social practices, Stalin’s cult had several sycophantic followers who engaged in exaggerated praises of Stalin. Lavrentiy Beria, chief of Soviet’s secret police, published a book dedicated to Stalin with the words: ‘To my dear, adored master, to the Great Stalin’ (Montefiore, 2004: 173). Another of Stalin’s closest associates, Lazar Kaganovich, gave an elaborate toast to Stalin describing him as the ‘steel founder of our socialist construction’ who had led the ‘socialist furnace without accidents and slow-downs’ and ‘smelted steel of a higher and unprecedented category’ (Davies, 2004: 40, 45). It was mandatory from 1935 onwards to speak of Stalin ‘only in the most glowing terms’, and sycophantic speeches were given even by his opponents (Davies, 1997: 148–149). Referring to Stalin as ‘the great leader’, ‘father of the people’, ‘the wise helmsman’, ‘the genius of our epoch’ and ‘the titan of the world revolution’ became commonplace (ibid.: 151).

Stalin’s cult was also resilient. In official images, he was treated as a symbol of the nation, downplaying private aspects of his persona (Pisch, 2016: 193), while unofficial depictions conversely tended to concentrate on personal details (Davies, 1997: 175). Neither type of images referenced his policies specifically, instead depicting him as various archetypes like the Father, Teacher or Saviour (Pisch, 2016: 22–23). The cult was likewise resilient from a social practice perspective, as Stalin’s charismatic authority did not rely on political success. The gulags and Stalin’s terror reign – sometimes labelled ‘the other Holocaust’ – started in the late 1920s and early 1930s (Cohen, 2011: 1–2), but was not linked to Stalin in the public imagination. One Soviet citizen recalled only positive memories from the 1930s, noting how ‘practically everything’ good was associated with the name Stalin. Injustices and incompetence were blamed on local authorities instead of Stalin. A group protesting the closure of their church in 1940 threatened to ‘go to *batiushka* Stalin, who allows us to keep our church, while all this is being done by local soviet power’ (Davies, 1997: 157–158). Evidently, the Soviet people were, consciously or not, overlooking Stalin’s shortcomings or wrongdoings, making the cult resilient from a social practice perspective.

The cult also had obvious religious parallels. Representationally, Stalin was often depicted in poses reminiscent of Christ and Orthodox saints, with his arm raised and palm forward in a pose suggesting a benediction. By the 1930s, icons associating Stalin with Christ were common in public life (Bonnell, 1997: 166, 168). Like with traditional icons, people covered or turned Stalin’s portrait away to block his spiritual presence and converse freely (Plamper, 2012: xvi). People would similarly pray and cross themselves in front of Stalin’s picture (Davies, 1997: 163). Ritualistic behaviour also occurred in public. Kremlin meetings were rituals attracting people from all over the Soviet Union. These theatrical meetings celebrated various events and were especially characterised by endless ovations (ibid.: 150). While only oligarchs were expected to stand up individually and proclaim their commitment to Stalin, the populace took part in collective rituals (Gill, 2021: 3). Everything from factory meetings or the beginning of the school year to the honouring of mothers who had produced many children were ‘public performances whose original intent became transformed into a celebration of Stalin’ (ibid.: 6). In sum, Stalin was depicted in a quasi-religious manner and certain rituals were connected to the cult.

### The Three Remaining Quadrants of the Model

If Stalin is the personality cult benchmark, what about the model’s three remaining quadrants? Pop icons differ from ‘normal’ celebrities as they have reached a fame level where they, like Jesus or Buddha, are known only by mononyms and ‘treated with the sort of respect traditionally reserved for religious figures’ (Till, 2010: 70). Beyoncé is, for instance, frequently depicted as resembling the Virgin Mary and referred to as ‘Beysus Christ’ by her loyal fanbase, the ‘Beyhive’ (Blyth, 2017). Pop icons can, however, not have personality cults as they lack legitimate power. Without legitimate power, the charismatic claim cannot be grounded in legitimate authority, thus making it incomplete. This lack of legitimate power is most evident in social practices as there are no signs of exaggerated praise by confirmation-biassed sycophantic followers or fans willing to follow any command. Although lack of personal authority could equally be a characteristic of the bottom right quadrant, pop icons typically have fewer cultlike manifestations on the representational dimension. Their images will especially be less widespread as they mainly circulate among fan groups. Pop icons’ irrelevance to people who are not fans reflects their lack of legitimate power. Their alleged charisma does not affect non-fans. A Metallica fan will, for instance, not be interested in depicting Beyoncé, while a Democrat might still disseminate or produce depictions of Trump – albeit probably not in a venerating way. Pop icons consequently belong in the bottom left quadrant, as they do not have cultlike tendencies in social practices and fewer of these manifestations representationally.

This only leaves the upper left and bottom right quadrant. The upper left concerns phenomena with personal authority evidenced by highly ritualistic behaviour and devoted followers but minimal or no sacrality in representations of the leader and no visible attempts at constructing charismatic authority. This combination is empirically unlikely as rituals require a symbol to revolve around. Márquez defines rituals as a practice where people interact while ‘focusing on some particular object or symbol’ (Márquez, 2018: 11). Leader representations are therefore prerequisites for establishing the grounds for cultish worship and rituals – and ultimately personality cults.

The bottom right quadrant reflects cultlike tendencies in representations but not in social practices. Although symbols of the leader are circulating, the images are not used actively by sycophants willing to follow the leader’s every command. This is more conceivable empirically, as the American civil religious ‘worship’ of several presidents fits within this category. Civil religion refers to ‘symbols of transcendence’ that unify and give meaning to the collective. The civil religious myths ‘represent a fusion of biblical and nationalistic imagery’ with American presidents being ‘priests and prophets’ (Fairbanks, 1981: 215, 223) – thus making the president the conveyer of the divine message without necessarily being the symbolic centre. Obama has been highlighted as an almost ideal–typical civil religious ‘priest’. He exemplified ‘the crux of civil religion’ in his Memorial Day 2008 speech, as his description of people’s willingness to sacrifice their life for the nation was based on a ‘common belief in the universal principles of life, liberty and the pursuit of happiness’ (Hammer, 2010: 281). Obama, occasionally dubbed ‘Obamessiah’, has been on magazine covers depicted in an angelic glow of light or as a Christ-like figure with a halo (Arogundade, 2017). Most iconic is the ‘Hope’ poster from his 2008 presidential campaign, depicting him in the same ‘three-quarters view’ as another civil religious priest, John F. Kennedy, and in the patriotic colour scheme of red, blue and white (Scott, 2017). While examples like these have made commentators label the Obama phenomenon a personality cult (‘The Obama Cult’, 2009), it only satisfies the representational dimension of the model – thus making it a civil religion. Charismatics are not just people who inspire. They perform ‘a particular social role’ as they use their charisma to legitimise their authority (Hendriks, 2017: 348).

Whether it is a civil religion or a personality cult thus depends on the level of charismatic authority. Flere (2007) argues that Josip Tito’s charismatic authority was the foundation of civil religion in Yugoslavia, thus implying that charismatic authority can exist side by side with, and even presuppose, civil religion. I, however, argue that this connection should be made with caution as high levels of charismatic authority will impede civil religion due to their different functions. Civil religion, unlike personality cults with a high level of charismatic authority, is not a form of personalised power or legitimation of the leader. Its function is to create societal cohesion, consequently making society the symbolic centre instead of the leader. The civil religious leader is symbolically elevated due to the office he or she represents. This limits the degree of charismatic authority that can be claimed since charismatic authority in civil religious societies is embedded in institutions and not the leader – he or she can, unlike the charismatic, not be the ‘revolutionary force’ working outside the confines of laws and societal norms. The mundane routinization and ‘steady developmental progression of established custom and legal/economic institutions’ evident from Weber’s rational-bureaucratic and traditional authority (Halley & Ordner, 2009: 59) are thus more compatible with civil religious worship as this form of leadership has no authority outside of the law.

Consequently, despite the Obama phenomenon’s resemblance to personality cults, civil religious worship is significantly different as it only exhibits one of the model’s two dimensions. Halley and Ordner (2009) argue that Obama has charismatic authority due to his status as a ‘polysemic icon’ who has garnered popular support from vastly different demographic groups, functioning as a uniting symbol (Halley & Ordner, 2009: 63). Obama has indeed (1) enjoyed widespread symbolic elevation in both official and unofficial depictions, (2) been presented as a symbol detached from his actual policies and (3) been depicted like a religious icon. While this places him high on the representational dimension, there are, however, no signs of exaggerated praises or sycophantic followers. While there are rituals, these are tied to society – Americans declare their allegiance to the flag, not the president. There are thus no signs of the symbolic elevation being translated into personalised power. In comparison, Trump demanding ‘total allegiance’ in his inaugural address foreshadowed his demands for personal loyalty from government officials. He equally neglected addressing the nation’s ‘moral standards and bedrock principles’ usually used to legitimise political authority. Obama referred to ‘freedom’ and ‘liberty’ 21 times in his inaugural address, while Trump only mentioned American freedoms once. In Trump’s regime, ‘there is no higher appeal’ (Carlson, 2018). Halley and Ordner’s use of charismatic authority to describe Obama is not necessarily misplaced, but it should be specified that he, unlike Trump, only exhibits low levels of charismatic authority and not the social practice dimension characteristic of personality cults.

### Trump, Putin and Ardern – Cults or Something In-between?

Both Putin and Trump enjoy widespread symbolic elevation on both dimensions. In addition to the Kremlin’s official pictures of a mostly shirtless Putin exploring nature, Putin frequently features on Western news media covers as, for instance, a Bond villain (Matthews, 2014) or a strategic chess player (‘Putin’s Endgame’, 2016). He similarly features in several internet memes, often showing him riding different animals or objects, or in artwork. Examples include Blue Noses’ critical depiction of him next to Russian cultural icon Alexander Pushkin and Christ (Goscilo, 2013: 26) or an exhibition depicting him doing the twelve labours of Hercules (Rosenberg, 2014). Although not all these venerate Putin, it demonstrates how the production and dissemination of his image has spread to different societal segments. As to social practices, there are numerous examples of sycophantic behaviour – like a Russian TV host exaggeratedly praising Putin for the seemingly insignificant act of catching a pencil rolling off the table (Ibbetson, 2021). More significantly, Patriarch Kirill, head of Russia’s Orthodox Church, described Putin’s rule as a ‘miracle of God’ (Bennetts, 2019), and speaker of the lower house of parliament, Vyacheslav Volodin, called Putin Russia’s main advantage in a world of challenges and threats (Kravchenko & Biryukov, 2020). Vladislav Surkov, formerly one of Putin’s closest aides, compared Putin’s model of state-building to the likes of Ivan the Great, Peter the Great and Lenin (Carroll, 2019). Even more hyperbolically, Chechen leader Ramzan Kadyrov called Putin a hero appointed by Allah and said people should ‘bow down before him’ (The Jamestown Foundation, 2007).

Trump frequently contributed to his own glorification through social media by, for instance, photoshopping his face onto Rocky Balboa’s body (Noor, 2019). Jon McNaughton, labelled the ‘unofficial artist of the Trump administration’ (‘Donald Trump’, 2019), has in a similar venerating fashion, for instance, depicted Trump as the fifth face on Mount Rushmore (McNaughton, n.d.). Other unofficial images range from multiple Time magazine covers (‘See every’, 2019), memes ridiculing his hairstyle and skin tone and a larger-than-life golden statue (Railton, 2021). These tendencies equally manifest themselves in social practices. Supporters even praise him for something that goes against their fundamental beliefs. Trump was called a hero for creating Covid-19 vaccines, although many of his supporters were fundamentally against vaccines (Nguyen, 2020). Sycophantic behaviour also extends to the government. Secretary of Energy, Rick Perry, compared Trump to biblical kings, calling him ‘the chosen one, sent by God to do great things’ (Lovett, 2019). This is particularly striking considering Perry, prior to Trump’s presidency, called Trump ‘a cancer on conservatism’ who would ‘lead the Republican Party to perdition’ (Glueck, 2015). Trump’s former campaign manager, Brad Parscale tweeted that ‘only God could deliver such a savior to our nation’, while former congresswoman Michele Bachman stated that there will never be ‘a more godly, biblical president again in our lifetimes’ (Mann, 2019). After passing a tax cut, former Vice-President Mike Pence praised Trump 14 separate times during a three-minute televised cabinet meeting. He stated that Trump has ‘signed more bills rolling back federal red tape than any president in American history’, and that he ‘spurred an optimism in this country that’s setting records’. The exaggeratedness is further emphasised, considering the tax cuts were only modest and temporary (Levitz, 2017).

Ardern, contrarily, does not enjoy widespread symbolic elevation. There is no evidence of official attempts at constructing charisma as she is neither posting cultlike images on social media like Trump nor posing for staged photos like Putin. Unofficial images of her are more prevalent as she is often depicted as Wonder Woman, and pop-cultural figures like *Star Wars*’ Princess Leia or à la the iconic feminist Second World War-poster ‘We Can Do It!’ (McNeilly, 2017). Although her image has been used for memes, it is less so than for Trump and Putin. There are also fewer signs of sycophantic behaviour. Although fans have called her ‘the best leader in the world’ (Loomes, 2020), she receives most praise from international media. French *Le Monde* labelled Ardern ‘a leader unlike any other’, and *Independent* called her a ‘beacon of hope in our tumultuous times’, making commentators state that her international praise overshadows her domestic popularity (Manhire, 2020). The international infatuation with Ardern is evident from the children’s book, *Taking the lead – How Jacinda Ardern wowed the world*, where she is the protagonist doing everything right and keeping all her promises (Hartwich, 2020). This black-and-white perspective dealing with absolutes, as customary in children’s books, parallels the exaggerated praises of Trump and Putin that equally portray them as infallible heroic figures. However, since exaggerated praise of Ardern is mainly witnessed internationally and not among government officials, the veneration does not permeate all layers of society – thus making it less pervasive on both dimensions than that of Trump and Putin.

Regarding the second parameter, Trump and Putin’s cults are resilient on both dimensions. Official images of Putin feature various action man-like wilderness expeditions, a theme equally recurring in most unofficial images. Even serious news media commenting on, for instance, Russia’s geopolitical strategies still depict Putin detached from his policies, instead focusing on his perceived unpredictability and cunningness, or juxtaposing him with Hollywood villains. In terms of social practice, various examples illustrate the cult’s resilience. As ‘little green men’ invaded Crimea in 2014, Putin initially denied any knowledge of them, stating they were not Russian soldiers but local Ukrainian citizens. Putin’s purpose of denying Russian presence in Ukraine was not to convincingly fool international observers, but to establish a ‘bond of willing ignorance with Russians’, who could demonstrate their loyalty by believing in Putin despite knowing he was lying. The more obvious the lie, the more suited it is for loyalty-signalling (Snyder, 2018: 163). A poll showed that when Putin publicly denied Russian presence in Crimea, the share of Russians who had come to trust him more in the last month rose from 42 to 44% (Clover, 2016: 18). Evidently, the obvious lie did not affect him negatively. Following the Crimean annexation, Russia faced sanctions which made Kremlin ban various European imports in retaliation. Coupled with the rouble losing over half its value, a disgruntled middle class would be expected. Polls, however, showed Putin’s rating continuously hovering between 85 and 90% (Dougherty, 2015). Even as particularly rural Russians lack essential public services like frequent trains or ambulances, Putin still avoids blame. A villager stated that ‘there are too many fraudsters behind Putin’s back’, and ‘if Putin knew, he wouldn’t ignore it’ – thus instead blaming people around Putin (Tsvetkova & Anin, 2014).

Although depictions of Trump are more varied and less centrally managed than Putin’s, their focus is still not on his policies. McNaughton tellingly classifies his paintings of Obama as political art, while his paintings of Trump are categorised as Americana – Trump is not just a politician, he embodies a nation and its values. In the evangelical documentary drama *The Trump Prophecy*, Trump’s abilities as a president or political viewpoints are equally considered irrelevant. The only thing that matters is that he was chosen by God (Burton, 2018). In terms of social practices, Trump has convincingly cemented himself as the one ‘cleaning up Washington’ and ‘the saviour of the common person’ to an extent where his supporters refuse to acknowledge when he does the opposite (Lempinen, 2020). At the Republican National Convention, one supporter applauded Trump for his ability to rise above criticisms without getting offended – despite him frequently calling opponents ‘nasty’, ‘loser’ or ‘dummy’. Others praised his handling of Covid-19 by banning travel to China, fast-tracking the vaccine and securing ‘record levels of testing’, although these measures’ effects were insignificant and characterised by repeated failures (Sargent, 2020).

Equally, a ‘quasi-religious doctrine of infallibility’ cultivated by Trump’s allies in Congress and the federal government indicates that blind loyalty extends into government. This entails that his allies frequently redefine the red line that cannot be crossed to uphold the perception that Trump can do no wrong (Serwer, 2019). When former Attorney General William Barr, during the investigation into Russian interference in the 2016 election, received documents showing Trump had engaged in what Barr had previously defined as obstruction of justice, he misrepresented the report’s conclusions to the public and dismissed it. Former Press Secretary Sean Spicer equally deliberately lied to the public when he criticised reports saying the crowd at Obama’s inauguration was bigger than Trump’s, although the truth was apparent (Thrush & Itzkoff, 2017). Additionally, Republican Senator Lindsey Graham stated he would support impeachment if it was established that Trump had engaged in quid pro quo. When it was established that Trump had, in fact, offered aid to Ukraine in exchange for publicly announcing that Joe Biden and his son were under investigation, it was framed by Republicans as the right approach to ensure no corruption had taken place in Ukraine before spending taxpayer money (Serwer, 2019). When Nixon’s involvement in the Watergate cover-up became clear in 1973, most Republicans supported impeachment, making his approval ratings drop to around 20%. Contrarily, Trump’s rating was about twice that of Nixon’s after his first impeachment, and he retained the support of most Republicans (Meyerson, 2019). The extent of his followers’ loyalty is, however, most evident in the storming of the Capitol, where Trump encouraged his supporters to make the lawmakers overturn the 2020 presidential election results (Elliott, 2021). Not only do Trump followers willingly overlook certain aspects of him, but they also follow his every command – even when it violates democratic or even legal behaviour.

Ardern’s alleged cult focuses more on her political success. Most depictions concentrate on her response to two major events during her tenure: the 2019 mosque massacre and Covid-19. She is, for instance, featured in a painting wearing a headscarf made of the New Zealand flag (‘Untitled’, 2019) and in a comic book equally wearing the headscarf referencing her empathetic response to the mosque attacks (‘Jacinda Ardern’, 2019). Her handling of Covid-19 is reflected in depictions of her as a saint with the coronavirus pathogen on her chest (Rayner, n.d.), or as an armour-clad knight fighting pathogens (Maley, 2020). Initially, Ardern’s alleged cult might seem resilient from a social practice perspective, as some argue that Ardern’s ‘halo’ has ‘blinded’ her fans to deeper problems (Cave, 2020) and that her popularity has ‘nothing to do with competent government administration and useful policies reliably delivered’. Especially according to international media, ‘Ardern has yet to put a foot wrong’ (Crabb, 2019). However, the word ‘yet’ reveals that although she is perceived to have governed admirably so far, she is not exempt from making future mistakes. She is not truly infallible. Only ‘hard core Jacindanistas’ remain delusional about her political accomplishments (Brook, 2020). Even an artist depicting Ardern as Wonder Woman, thus participating in the ‘cult’, stated that she does not put her ‘on a pedestal’ (Bhatia, 2020). Loyal Labour voters likewise state that while Ardern is ‘really nice’, she has not delivered on her promises. Her international superstar status is increasingly seen as ‘good marketing’, leading some to conclude that ‘the gloss has definitely come off Ardern’ (Roy, 2020). Ardern acknowledges this by stating that popularity fluctuations reflect that she takes on ‘big issues’. After her applauded response to the terrorist attacks, she peaked at 51%, dropping to 41% 3 months later (Cooke, 2019). Recent polls show a ‘personal popularity crash’ with approval dropping 15 points (Coughlan, 2021). This follows the lukewarm reception of the housing reform, further indicating that Ardern’s popularity relies on her political success.

Regarding the third parameter, religious parallels are evident in the representations of all three politicians. Putin is depicted à la Russian icons on a golden background with a halo (d’Amora, 2015), as an angel extending his hand to bless the people of Saint Petersburg (Jackson, 2011), and Saint George slaying the dragon on a collectable silver coin (‘Victorious Saint George’, n.d.). Trump is similarly depicted like a Russian Orthodox icon wearing a red cloak and golden halo (Gauthier, 2016), and it is even possible to buy, for instance, a t-shirt or tapestry with an icon-like Trump as the ‘Lord and Savior’ holding a lamb on the online marketplace Redbubble (TrumpJesus, n.d.). Ardern is likewise canonised as ‘a living saint’ in a portrait clad in red with a halo around her head (Rayner, n.d.), or in the style of the Virgin Mary (Jewelia Howard Art, n.d.).

Both Trump and Putin’s cults equally have religious parallels in social practices. When Putin unexpectedly stopped in Izborsk, the path he had walked through town was marked – where he had bought cucumbers, drunk from the fountain and touched a tree and made a wish. Likewise, Magnitogorsk Museum exhibited the overalls he had worn under his visit. Visiting these locations becomes a matter of pilgrimage (Goscilo, 2013: 12). Another ritual is his annual call-in show broadcasted on state television. The show features Putin taking questions and complaints from the public. Afterwards, news media report how Putin fixed all issues addressed on the show – from decaying schools to giant potholes (Higgins, 2019).

Similarly, Trump’s cabinet meetings have been described as a ‘bizarre spectacle’, excessively commending Trump and his accomplishments (Nguyen, 2017). In addition to serve as public loyalty-signalling, the way Trump ceremoniously went around the table, instructing each cabinet member to praise him one by one, emulated a ritual. Trump’s rallies also resemble rituals creating a sense of belonging to an exclusive group of the initiated. Membership of this congregation is shown by bringing the thumb and index finger together in the ‘OK’ gesture – a coded message allegedly representing the letters ‘WP’ for ‘white power’. Like disciples of Christ, supporters follow Trump from rally to rally. The rallies themselves are considered a church for the like-minded, the followers recognised by the red hats Trump throws at the crowd, ‘giving of himself’ (Sharlet, 2020). This thus parallels the ritual of consuming sacramental bread and wine as the body of Christ, reminding the followers of their leader’s sacrifice.

Rituals are, however, not prevalent in Ardern’s alleged cult. During the Covid-19 lockdown in 2020, Ardern did weekly updates from her home, covering everything from serious political matters to her daughter’s naptime in a style reminiscent of ‘a conversation you might hear over dinner’ (Cave, 2020). Her live videos on Facebook added an interactive element with people commenting and getting immediate reactions from her. Although this seems on par with Putin’s call-in show, rituals around Ardern are not commonplace. Additionally, most rituals surrounding Trump and Putin originated at the grassroot level. This shows the existence of a community of like-minded people actively seeking out participation in the cult and engaging in genuine worship of the leader – something that Ardern’s ‘cult’ shows no sign of.

## Conclusion

This paper introduced a model for distinguishing between popularity and personality cults based on three parameters covering a representational and social practice dimension. Putin, Trump and Ardern were used to illustrate the model’s ability to categorise phenomena with different degrees of charisma. The analysis shows that while Trump and Putin belong in the domain of personality cults, Ardern’s alleged cult does not have a social practice dimension, as the few cultlike tendencies are strictly representational. The phenomenon surrounding Ardern more likely marks the beginning of a civil religion being formed in New Zealand. Like Obama came to represent hope, Ardern embodies compassion in a way that unites the country in times of need.

While the examples illustrate the use of the model, there is a need for a comprehensive analysis to offer more conclusive statements regarding the differences in the degree of charisma between Ardern, Putin and Trump. Although not covered by the model, this would include looking into whether there are differences between presidential and parliamentarian systems – and if so, what these differences are. Furthermore, although the proposed model distinguishes popular politicians from personality cults, more research is needed on how, for instance, Stalin’s and Putin’s personality cults differ. The difference between modern and postmodern personality cults cannot simply be reduced to whether they are patricentric. Putin’s personality cult differs from his predecessors as it is ‘inherently polysemantic, highly mobile, and easily individualized’ (Cassiday & Johnson, 2013: 40). These differences arise due to modern communicative technology and the capitalist marketplace making it difficult to establish the same societal control as previously (ibid.: 49). Future research thus needs to examine how particularly new communication forms, like social media, affect the dynamics of personality cults.
